# Diagnosis of early stage knee osteoarthritis based on early clinical course: data from the CHECK cohort

**DOI:** 10.1186/s13075-021-02598-5

**Published:** 2021-08-19

**Authors:** Qiuke Wang, Jos Runhaar, Margreet Kloppenburg, Maarten Boers, Johannes W. J. Bijlsma, Sita M. A. Bierma-Zeinstra, the CREDO expert group, N. E. Aerts-Lankhorst, R. Agricola, A. N. Bastick, R. D. W. van Bentveld, P. J. van den Berg, J. Bijsterbosch, A. de Boer, M. Boers, A. M. Bohnen, A. E. R. C. H. Boonen, P. K. Bos, T. A. E. J. Boymans, H. P. Breedveldt-Boer, R. W. Brouwer, J. W. Colaris, J. Damen, G. Elshout, P. J. Emans, W. T. M. Enthoven, E. J. M. Frölke, R. Glijsteen, H. J. C. van der Heide, A. M. Huisman, R. D. van Ingen, M. L. Jacobs, R. P. A. Janssen, P. M. Kevenaar, M. A. van Koningsbrugge, P. Krastman, N. O. Kuchuk, M. L. A. Landsmeer, W. F. Lems, H. M. J. van der Linden, R. van Linschoten, E. A. M. Mahler, B. L. van Meer, D. E. Meuffels, W. H. Noort-van der Laan, J. M. van Ochten, J. van Oldenrijk, G. H. J. Pols, T. M. Piscaer, J. B. M. Rijkels-Otters, N. Riyazi, J. M. Schellingerhout, H. J. Schers, B. W. V. Schouten, G. F. Snijders, W. E. van Spil, S. A. G. Stitzinger, J. J. Tolk, Y. D. M. van Trier, M. Vis, V. M. I. Voorbrood, B. C. de Vos, A. de Vries

**Affiliations:** 1 Department of General Practice, Erasmus MC University Center Rotterdam, Rotterdam, The Netherlands; 2 Department of Rheumatology, Leiden University Medical Center, Leiden, The Netherlands; 3 Department of Epidemiology & Biostatistics, Amsterdam UMC, Amsterdam, The Netherlands; 4 Department of Rheumatology and Clinical Immunology, University Medical Centre Utrecht, Utrecht, The Netherlands; 5 Department of Orthopaedics, Erasmus MC University Center Rotterdam, Rotterdam, The Netherlands

**Keywords:** Early diagnosis, Early clinical course, Knee osteoarthritis, CHECK cohort

## Abstract

**Background:**

Early diagnosis of knee osteoarthritis (OA) is important in managing this disease, but such an early diagnostic tool is still lacking in clinical practice. The purpose of this study was to develop diagnostic models for early stage knee OA based on the first 2-year clinical course after the patient’s initial presentation in primary care and to identify whether these course factors had additive discriminative value over baseline factors.

**Methods:**

We extracted eligible patients’ clinical and radiographic data from the CHECK cohort and formed the first 2-year course factors according to the factors’ changes over the 2 years. Clinical expert consensus-based diagnosis, which was made via evaluating patients’ 5- to 10-year follow-up data, was used as the outcome factor. Four models were developed: model 1, included clinical course factors only; model 2, included clinical and radiographic course factors; model 3, clinical baseline factors + clinical course factors; and model 4, clinical and radiographic baseline factors + clinical and radiographic course factors. All the models were built by a generalized estimating equation with a backward selection method. Area under the receiver operating characteristic curve (AUC) and its 95% confidence interval (CI) were calculated for assessing model discrimination. Delong’s method compared AUCs.

**Results:**

Seven hundred sixty-one patients with 1185 symptomatic knees were included in this study. Thirty-seven percent knees were diagnosed as OA at follow-up. Model 1 contained 6 clinical course factors; model 2: 6 clinical and 3 radiographic course factors; model 3: 6 baseline clinical factors combined with 5 clinical course factors; and model 4: 4 clinical and 1 radiographic baseline factors combined with 5 clinical and 3 radiographic course factors. Model discriminations are as follows: model 1, AUC 0.70 (95% CI 0.67–0.74); model 2, 0.74 (95% CI 0.71–0.77); model 3, 0.77 (95% CI 0.74–0.80); and model 4, 0.80 (95% CI 0.77–0.82). AUCs of model 3 and model 4 were slightly but significantly higher than corresponding baseline-factor models (model 3 0.77 vs 0.75, *p* = 0.031; model 4 0.80 vs 0.76, *p* = 0.003).

**Conclusions:**

Four diagnostic models were developed with “fair” to “good” discriminations. First 2-year course factors had additive discriminative value over baseline factors.

**Supplementary Information:**

The online version contains supplementary material available at 10.1186/s13075-021-02598-5.

## Background

Early diagnosis of knee osteoarthritis (OA) is important in managing this disease, as it helps open a ‘treatment window’ for early interventions which could positively modify the disease course [1–4]. Nowadays, such an early diagnostic tool is still lacking in clinical practice.

Individuals who will develop established OA could be considered as being at early stage of OA in the years prior to the diagnosis of established OA. With applying multivariable prediction models, early diagnostic algorithms can be built by connecting multiple present predictors with the future occurrence of established OA [5, 6]. A few knee OA models, including clinical manifestations together with imaging features [7, 8] or laboratory biomarkers [9–13], have been proposed for building (early) predictive configurations. As no gold standard has been established for diagnosing knee OA in clinical practice (as opposed to classification criteria intended for studies), these models were built using heterogeneous outcomes; American College of Rheumatology criteria based clinical OA [12, 13], persistent knee pain [14], or (incident) radiographic OA [7–11]. A better way to minimize the “gap” between “research classification criteria” and “unknown gold criteria” is to obtain a clinical expert consensus-based diagnosis, as we have done in a previous study [15].

All of the above models were based on baseline factors only; none evaluated the diagnostic value of the early clinical course. Knee OA progression has been reported to follow a pattern of inertia [16], which means knees with recent progress will continue to progress in the future and are more likely to develop into established OA. In turn, these knees should be considered as being at early stage OA at this moment. Besides, a “wait-and-see” policy is frequently applied by clinicians while treating knee complaints with a recent onset and with mild symptoms, suspected but not confirmed for knee OA [17, 18]. Repeated consultations are quite common for such a chronic disease. Hence, early clinical course data of knee OA is often clinically accessible.

In our previous study, we built early diagnostic models for clinical expert consensus-based diagnosis by including baseline factors [15]. In this study, we aimed to use the first 2-year course factors, as well as the combinations of baseline and course factors, to build diagnostic models for the same expert diagnosis. Additionally, we aimed to see whether course factors had additive discriminative value over baseline factors.

## Methods

### Data source and patients

We obtained patient data from the CHECK cohort (a longitudinal cohort study of patients with knee or hip complaints suspect for early stage OA, followed for 10 years) [19, 20]. The inclusion criteria of CHECK cohort were (1) non-traumatic knee or hip pain or stiffness, (2) aged 45–65 years old, (3) no previous consultation, or the first consultation with a general practitioner within 6 months before inclusion. The CHECK cohort excluded the patients if the complaints could be explained by other diseases than OA. Patients in the CHECK cohort fulfilled questionnaires and got physical and radiographic examinations at baseline, 2, 5, 8, and 10 years. See more details in other papers [19, 20].

This study included all the knees with reported symptoms at baseline and had data available throughout the 10 years. If the patient reported bilateral knee symptoms at baseline, both knees would be included.

### First 2-year course factors and definitions

We collected identical factors at baseline and 2-year follow-up, including body mass index (BMI, kg/m^2^); bilateral knee pain (yes/no); physical examinations (presence of joint line tenderness, bony swelling at the joint margins, warmth, effusion, crepitus, patellofemoral joint grinding, restricted/painful flexion/extension, Heberden nodes); Western Ontario and McMaster Universities Osteoarthritis Index (WOMAC) questionnaires [21] (we selected knee OA related items, includes 5 individual items for pain, 6 for function and 1 for knee stiffness; all are graded from 0 to 4), and radiographic items (medial/lateral tibiofemoral osteophytes, medial/lateral tibiofemoral joint space narrowing (JSN), patellofemoral osteophytes, patellofemoral JSN and tibiofemoral joint angle). We defined restricted/painful flexion as maximal knee flexion ≤ 115° or pain at knee flexion; restricted/ painful extension as an extension deficit ≥ 1° or pain at knee extension. We measured tibiofemoral joint angle on standardized weight-bearing posterior-anterior radiographs using Knee Images Digital Analysis (KIDA) software [22]. Trained readers scored the radiographic items according to Kellgren & Lawrence criteria [23] via a centralized reading of standardized posterior-anterior and lateral radiographs. Readers got information on the sequence of images but were blinded to the clinical information [19].

We defined the first 2-year course factors according to the factors’ change over this period. BMI change greater than 5% was considered as clinically relevant [24, 25], so we code course factor for BMI into decrease (BMI decreased ≥ 5%), increase (BMI increased ≥ 5%), and stable. For bilateral knee pain and physical examination items, we code each course factor into negative at both time points (baseline and 2-year follow-up), positive at either time point, and positive at both time points. For WOMAC individual items, osteophyte and JSN, we code course factors into three categories by the changes in severity: decrease (severity decreased one grade or more), increase (severity increased one grade or more), and stable. We chose the “one grade” as the threshold mainly based on prior knowledge that ‘one grade’ is considered as a minimal detectable difference in the WOMAC questionnaire [21, 26] and Kellgren & Lawrence grading system [23]. According to the previous literature, tibiofemoral joint angle change of less than 2° should be considered as measurement error [22]. Hence, we code the course factor for joint angle into decrease (angle decreased ≥ 2°), increase (angle increased ≥ 2°), and stable.

Knowing that few patients (1%) presented bony swelling, joint warmth, and joint effusion at both time points, we incorporated these patients into the category of positive at either time point. Similarly, few patients (1–3%) presented decreased severity in radiographic items (except tibiofemoral joint angle), thus we incorporated these into the stable category.

### Outcome factor

We used the clinical expert consensus-based diagnosis as the outcome factor. Our previous studies described the process in detail [15, 27]. Briefly, we recruited both general practitioners and secondary care physicians to evaluate each knee’s longitudinal (from 5- to 10-year follow-up) clinical and radiographic data. Clinical experts made the final diagnosis for each knee of whether clinically relevant knee OA developed during follow-up based on consensus. No formal definition of clinically relevant knee OA was provided to the clinicians; they were instructed to use their own clinical expertise to judge this. The final diagnosis was made upon agreement by clinicians (intraclass correlation coefficient 0.908; 95% confidence interval (CI), 0.821 to 0.965) [15] and for each knee, the final diagnosis could be one of the following options: OA, no OA, and uncertain.

### Statistics

We checked missing data of baseline and 2-year follow-up factors and replaced them by multiple imputation (created 50 datasets, 49% cases had incomplete data, but only 2 variables had more than 10% missing values). Next, we created course factors for each knee.

During the model building process, we firstly excluded knees that were diagnosed as “uncertain.” We did not calculate the formal sample size but were sure to meet the rule of thumb for at least 10 OA knees per predictor. We adopted the same models (contains baseline factors only) as we developed in the previous study as the baseline-factor models for this study [15]. For building course-factor models, we used the same stepped approach as for our baseline-factor models. First, we build a model by including clinical course factors only (model 1) and then including both clinical and radiographic course factors (model 2). Since the two knees from the patients with bilateral complaints would share the same personal data (i.e., age, sex, and BMI) and might have correlated measurement results, we treated the data of the two knees as repeated measures within one person. To adjust for repeated measures, we applied generalized estimating equation (GEE) with a backward selection method (*P* > 0.1 removal) to build the models. In this way, final models can be used for calculating the probabilities of individual knees. With treating the category of stable or negative at both time points as reference, we incorporated the other two categories into the reference category if tested insignificant (*P* > 0.1).

Finally, we added factors of the final model 1 into our baseline clinical-factor model (developed in our previous study [15]). We built this combined clinical model (model 3) by the identical backward selection method as described above. Similarly, we added the final model 2 factors into the baseline clinical and radiographic factor model and got the combined clinical and radiographic model (model 4).

We presented all model factors as pooled odds ratios (OR) and 95% CI, and tested model discrimination via the receiver operating characteristic curve. Pooled area under the curve (AUC) and its 95% CI were calculated. To identify whether course factors have additive discriminative value over baseline factors, we compared AUC values of model 3 and model 4 with those of the two corresponding baseline-factor models using the method of Delong et al. [28]. To evaluate each factor's contribution in the 4 models, we continued backward selection and removed the factor (with the highest *p* value) step by step until the last one. AUC was calculated for each step.

We internally validated all the models by estimating model calibration and over-fitting [29]. We tested model calibration via calibration plot and Hosmer and Lemeshow statistics. *P* > 0.05 of Hosmer and Lemeshow test indicates good calibration. We detected model over-fitting by bootstrapping 1000 samples from the derivation dataset (with replacement) [29]. The amount of optimism was evaluated according to the change in AUC.

We performed sensitivity analysis for the 4 models by including ‘uncertain’ knees into the dataset, and assessed model discriminations when treating ‘uncertain’ knees as OA knees and as no OA, respectively.

Model building, discrimination, and sensitivity analysis were performed with software SPSS version 25.0 (IBM, Chicago, USA). AUC comparison and internal validation were performed with R software version 3.6.1. Development and reporting these models followed TRIPOD (Transparent Reporting of a multivariable prediction model for Individual Prognosis Or Diagnosis) guidance (see Additional file 1 TRIPOD checklist) [29].

## Results

### Patients and factors

Seven hundred sixty-one patients with 1185 symptomatic knees were included in this study. Nine hundred forty-eight (79%) were female; the mean (SD) age is 56 (5) years. Characteristics, pooled after multiple imputation, of baseline and course factors, are presented in Table 1. Four hundred thirty-eight (37%) knees were diagnosed as OA, 532 (45%) were as no OA, and 215 (18%) were as “uncertain” in the final diagnosis.

**Table 1:** Characteristics of baseline and first 2-year course factors

Factors	Baseline, presented positive ***N*** (%)	Decrease/negative at both time points ***N*** (%)	Stable/positive at either time point ***N*** (%)	Increase/positive at both time points ***N*** (%)
**BMI**	26.3 (4.2)^c^	154 (13)	892 (75)	139 (12)
**Bilateral knee pain**	848 (72)	152 (13)	447 (37)	586 (50)
**Joint line tenderness**	528 (45)	541 (45)	390 (33)	254 (22)
**Bony swelling**	43 (4)	1092 (92)	88 (7)	5 (1)
**Joint warmth**	48 (4)	1103 (93)	77 (6)	5 (1)
**Joint effusion**	80 (7)	1042 (88)	137 (11)	6 (1)
**PF joint crepitus**	533 (45)	500 (42)	319 (27)	366 (31)
**PF joint grinding**	370 (31)	727 (61)	320 (27)	138 (12)
**Restricted/painful flexion**	327 (30)	359 (30)	458 (39)	368 (31)
**Restricted/painful extension**	606 (51)	706 (60)	360 (30)	119 (10)
**Heberden nodes**	604 (52)	384 (32)	270 (23)	531 (45)
**WOMAC pain**^a^				
Walking	202 (17)	273 (23)	664 (56)	248 (21)
Standing	249 (21)	248 (21)	652 (55)	285 (24)
Stairs	569 (48)	332 (28)	592 (50)	261 (22)
Night	379 (32)	380 (32)	545 (46)	260 (22)
Rest	308 (26)	320 (27)	628 (53)	237 (20)
**WOMAC function**^a^				
Descending	355 (30)	332 (28)	593 (50)	260 (22)
Ascending	486 (41)	332 (28)	569 (48)	284 (24)
Rising	438 (37)	320 (27)	640 (54)	225 (19)
Standing	225 (19)	237 (20)	675 (57)	273 (23)
Walking	166 (14)	236 (20)	712 (60)	237 (20)
Sitting	178 (15)	263 (22)	687 (58)	235 (20)
**WOMAC morning stiffness**^a^	590 (50)	379 (32)	545 (46)	261 (22)
**Medial TF osteophyte**^b^	130 (11)	20 (2)	950 (80)	215 (18)
**Lateral TF osteophyte**^b^	189 (16)	10 (1)	904 (76)	271 (23)
**Medial TF JSN**^b^	73 (6)	24 (2)	960 (81)	201 (17)
**Lateral TF JSN**^b^	11 (1)	35 (3)	1045 (88)	105 (9)
**PF osteophyte**^b^	130 (11)	23 (2)	1055 (89)	107 (9)
**PF JSN**^b^	36 (3)	9 (1)	955 (81)	221 (18)
**TF angle**	− 1.8 (1.8)^c^	305 (26)	761 (64)	119 (10)

*BMI*, body mass index; *PF joint*, patellofemoral joint; *WOMAC*, Western Ontario and McMaster Universities Osteoarthritis Index; *TF*, tibiofemoral; *JSN*, joint space narrowing; percentages are calculated by dividing total number of knees (1185)

^a^All the WOMAC items have 5 grades (0 to 4). Patient graded at 2 or more were treated as positive presentation at baseline

^b^Radiographic items were graded according to Kellgren & Lawrence criteria with 5 grades (0–4), and grade 2 or more was treated as positive presentation at baseline

^c^Presented as mean (standard deviation)

### Models

Six clinical course factors were retained in model 1, and 9 (6 clinical and 3 radiographic) course factors were retained in model 2. Pooled OR are presented in Table 2. In both model 1 and model 2, worsening of clinical or radiographic signs over the first 2 years, except the course of restricted/ painful extension, indicated a higher probability of early stage knee OA.

**Table 2:** Four models and pooled OR (odds ratios) for diagnosing early stage OA

	Model 1	Model 2	Model 3	Model 4
	Pooled OR (95% CI)	Pooled OR (95% CI)	Pooled OR (95% CI)	Pooled OR (95% CI)
**Baseline factors**				
**BMI**			1.12 (1.08–1.17)	1.10 (1.06–1.15)
**Gender (female)**			1.60 (1.10–2.33)	1.67 (1.14–2.46)
**WOMAC function descending**				
Positive vs. negative^a^			1.94 (1.35–2.78)	1.85 (1.29–2.64)
**WOMAC function rising**				
Positive vs. negative^a^			1.56 (1.10–2.21)	
**WOMAC stiffness**				
Positive vs. negative^a^			1.56 (1.13–2.16)	1.94 (1.41–2.67)
**Joint effusion**				
Positive vs. negative ^b^			4.36 (2.35–8.12)	
**Medial TF JSN**				
Positive vs. negative^b^				3.70 (2.07–6.63)
**Course factors**^c^				
**Joint line tenderness**				
Positive at either time point	1.58 (1.16–2.17)	1.59 (1.15–2.19)	1.57 (1.13–2.19)	1.64 (1.17–2.31)
Positive at both time points	2.28 (1.58–3.29)	2.20 (1.51–3.22)	1.83 (1.24–2.70)	1.95 (1.31–2.91)
**Joint effusion**				
Positive at either time point	3.28 (2.07–5.20)	2.98 (1.84–4.81)		3.00 (1.82–4.95)
**Restricted/painful flexion**				
Positive at either time point	1.49 (1.09–2.04)	1.36 (0.99–1.88)		
Positive at both time points	2.54 (1.51–4.27)	2.35 (1.38–3.99)		
**Restricted/painful extension**^d^				
Positive at either time point	0.74 (0.55–0.99)	0.69 (0.51–0.94)	0.70 (0.51–0.96)	0.66 (0.48–0.91)
**PF joint crepitus**				
Positive at either time point	1.44 (1.03–2.01)	1.45 (1.03–2.03)		
Positive at both time points	1.87 (1.34–2.62)	1.87 (1.32–2.64)	1.85 (1.34–2.55)	1.68 (1.21–2.34)
**WOMAC function descending**^e^				
Increase	1.66 (1.19–2.32)	1.81 (1.29–2.55)	2.07 (1.45–2.96)	2.30 (1.59–3.32)
**Medial TF osteophyte**				
Increase		1.68 (1.15–2.46)		1.59 (1.06–2.40)
**Lateral TF osteophyte**				
Increase		2.08 (1.46–2.95)		1.84 (1.26–2.70)
**PF JSN**				
Increase		2.59 (1.49–4.51)		2.45 (1.37–4.39)
**Pooled model intercept**	− 1.16	− 1.43	− 4.81	− 4.76

*BMI*, body mass index; *WOMAC*, Western Ontario and McMaster Universities Osteoarthritis Index; *TF*, tibia femoral; *JSN*, joint space narrowing; *PF*, patellofemoral joint

Model 1, included clinical course factors only; model 2, included clinical and radiographic course factors; model 3, clinical course factors + clinical baseline factors; model 4, clinical and radiographic course factors + clinical and radiographic baseline factors

^a^All the WOMAC items have 5 grades (0 to 4). Patient graded at 2 or more were treated as positive presentation at baseline, negative knees were treated as reference

^b^Radiographic items were graded according to Kellgren & Lawrence criteria, and grade 2 or more was treated as positive; negative knees were treated as reference

^c^Category of negative at both time points or stable was treated as reference. Few patients (1%) presented joint effusion at both time points; we incorporated these patients into the category of positive at either time point before statistical analysis. Few patients (1–3%) presented decreased severity in radiographic items, and we incorporated them into the stable category before statistical analysis

^d^The category of positive at both time points was not significant and incorporated into the reference category

^e^The decrease category was not significant and incorporated into the reference category

Six baseline clinical factors combined with 5 clinical course factors were retained in model 3, and 5 (4 clinical and 1 radiographic) baseline factors combined with 8 (5 clinical and 3 radiographic) course factors were retained in model 4. Pooled OR are presented in Table 2. In both model 3 and model 4, more severe baseline status combined with worsening of clinical or radiographic signs over the first 2 years, except the course of restricted/painful extension, indicated a higher probability of early stage knee OA.

**Fig. 1:**
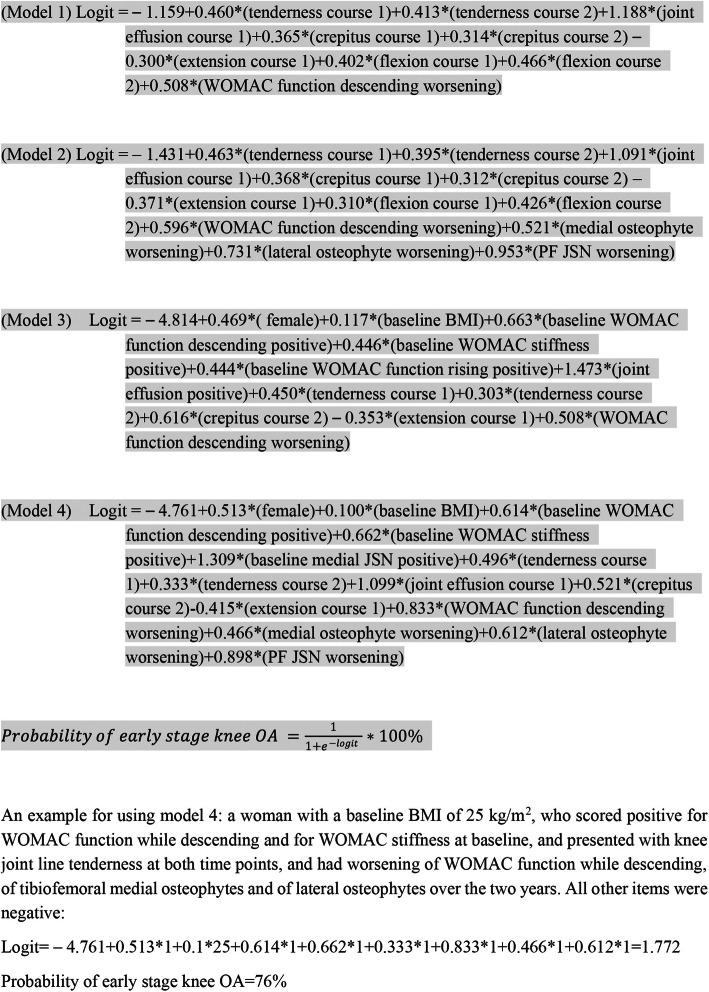
Final equations for the four models. For binary outcome, we use the link function of logit. Personal probability of early knee OA can be calculated by using one of the following formulas. Course 1, positive at either time point; course 2, positive at both time points; PF, patellofemoral; JSN, joint space narrowing

Four final model equations for calculating individual probability are presented in Fig. 1.

### Model discrimination and factors’ contributions

Model 1 and model 2 were able to discriminate between patients with and without knee OA with pooled AUC of 0.70 (95% CI 0.67–0.74) and 0.74 (95% CI 0.71–0.77), respectively. Each factor’s contribution is presented in the Additional file 1 figure. The course of joint line tenderness was the most significant factor in both model 1 and model 2.

The pooled AUC of model 3 and model 4 were 0.77 (95% CI 0.74–0.80) and 0.80 (95% CI 0.77–0.82), respectively. Model discriminations were significantly higher than their corresponding baseline-factor models (model 3, 0.77 vs 0.75, *p* = 0.031; model 4, 0.80 vs 0.76, *p* = 0.003). Each factor’s contribution to model 3 and model 4 is presented in the Additional file 1 figure. Baseline BMI was the most significant factor in both model 3 and model 4.

### Internal validation and sensitivity analysis

**Fig. 2:**
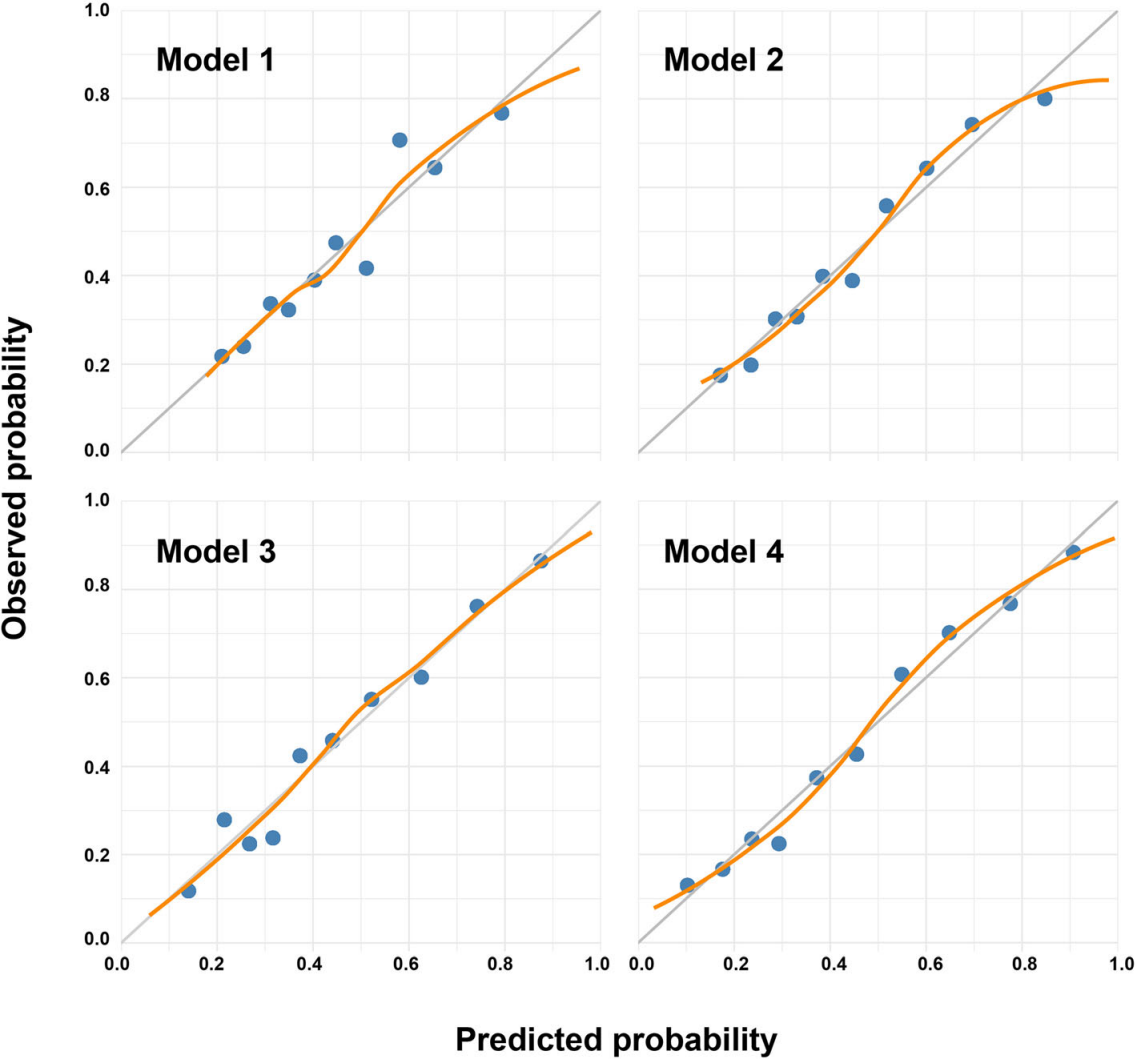
Calibration plots of the four models. Blue points represent data points of mean predicted against mean observed within certain range of predicted probability. Orange line represents a regression smoother through data points. Gray line represents perfect calibration

Both model 1 and model 2 presented good internal calibration (model 1, *p* = 0.24; model 2, *p* = 0.40) (Fig. 2) and were detected with no over-fitting when rounded to two decimals (Additional file 1: table 1). Sensitivity analysis showed minimal reduction (3% to 5%) of AUCs while incorporating “uncertain” knees into the dataset for both model 1 and model 2 (Additional file 1: table 2).

Both model 3 and model 4 presented good calibration (model 3, *p* = 0.78; model 4, *p* = 0.36) (Fig. 2). No over-fitting was found in model 3, and only a minimal optimism of AUC (1%) was detected in model 4 (Additional file 1: table 1). Sensitivity analysis showed minimal reduction (4% to 6%) of AUCs while incorporating “uncertain” knees into the dataset for both model 3 and model 4 (Additional file 1: table 2).

## Discussion

This study showed that information on the early clinical course can help to diagnose early stage knee OA. Both models with only course factors included presented “fair” discriminations and good internal validations. Adding these identified course factors into baseline models, we found the two combined models had significantly better discriminative abilities than the baseline models. However, the improvements in AUC should be considered as small and further studies are needed for evaluating the clinical relevance. Baker et al. reported that interpretation of a small increase in AUC should be made based on balancing benefits and costs of obtaining new factors; even an increase of 0.02 in AUC by additional factors was demonstrated worthwhile in their study [30].

The principal motivation for this study was to build implementable diagnostic tools for early stage knee OA. For this purpose, our study was designed to have the following strengths: first, our models were built among a large population who were in suspicion of early stage knee OA and began to look for medical care in primary care [19]. Comparing to other well-known early stage knee OA cohorts, such as the osteoarthritis initiative (incident OA subgroup) [31], MOST [32], and CASK [33] cohorts, the population in CHECK presents an even earlier stage with milder structural damage. Therefore, the first 2-year course identified in this study should be considered as early clinical course. Second, our models used diagnoses of real clinical experts (experienced general practitioners and secondary care physicians) as the reference standard, obtained via a pre-designed protocol. Most diagnoses of prior knee OA models were based on radiographic assessments, equaling (incident) radiographic knee OA [7–11]. However, not all of these radiographic OA would be diagnosed as OA in real practice; in the CHECK cohort, the overlap between radiographic knee OA and the expert diagnosis was only 59% [15]. Third, the factors in our models are clinically implementable assessments. As early diagnosis is mainly done in primary care, where the use of radiography to diagnose OA is discouraged, our model 1 and model 3 can be used. Model 2 and model 4 can be implemented in clinical settings where radiographs are available. In contrast, models that apply novel factors such as (quantified) MRI features and laboratory (genetic) biomarkers [7–13], are costly and not applicable in daily clinical practice. In addition, the AUCs of such models have ranged from 0.72 to 0.83 [7–13], fully comparable with our results obtained with routine and low cost procedures.

Theoretically, the individual probability of early stage knee OA can be calculated via inputting personal attributes into our model equations. The additive discriminative value of course factors indicates that the 2-year follow-up based early diagnosis should be more accurate than using baseline data only. On the other hand, based on the equations, it makes more sense to apply these models to the patients with worsening conditions within the first 2 years, since these patients are more likely to result in higher probabilities and need treatment. Furthermore, these findings raise a new strategy for selecting predictors. Future studies on building prediction/diagnostic models for knee OA could also take a period of disease course into account.

This study presented the first step of establishing early diagnostic criteria based on patients’ first 2-year clinical course, but certainly, further studies are warranted before implementing these models in real practice. There are probably too many factors in our final models, especially model 3 and model 4, which increases the difficulty of implementing in real practice. Making models more concise seems necessary. According to each factor’s contribution in our models, it should be feasible to decrease the number of factors in model 3 and model 4 to a maximum of 6 or 7 with AUC values around 0.75 (AUC of 0.75 or above indicates clearly useful discrimination [34]). Further evaluations on each factor’s clinical implications as well as the costs and benefits are required before deciding which factors to be removed. Meanwhile, model external validation is an essential step as it evaluates the reliability of applying models in other populations. Moreover, to establish clinically practical diagnostic criteria, a probability threshold for ruling in and ruling out early stage knee OA is needed, after which diagnostic measures including predictive values, sensitivity, and specificity can be assessed.

In general, model factors indicated that knees with early deterioration are more likely to have early stage knee OA, which is mostly consistent with the results of other studies [16, 35, 36]. It is notable that one course factor, restricted/painful extension, was inversely related to clinically relevant knee OA in our models. A similar phenomenon was found in another study [37] whose model presented dyslipidemia and a family history of premature coronary artery disease was a protective factor for all-cause mortality. This was explained as caused by an unmeasured confounder of lipid-lowering medications usage in these patients. In this study, the patients with restricted/painful joint extension (presented at either time point) probably get some unmeasured but effective interventions as well, such as physical therapy [38, 39]. We incorporated this factor in our model mainly for its additive discriminative ability, but this inverse relationship needs to be externally validated.

There are several limitations in this study. First, misclassification bias cannot be ruled out when dealing with course factors, especially in radiographic factors. As we can see, some knees were found to have milder structural features after the 2 years, most of which should be considered as misclassifications (measurement errors). Given the low rates and these variables were created independent from the outcome, it should be considered as non-differential misclassification bias and has a very limited impact on model estimates. Second concern is that our models are based on the first 2-year follow-up, which means the model makes an early diagnosis 2 years after baseline. The cost of this time delay is unknown. Since our baseline assessment was at the patient’s first consultation for knee complaint and the CHECK cohort was proven to include patients at an early stage, we assume ‘wait and see’ or ‘inconclusive diagnosis’ together with some symptomatic treatments in the 2 years is justifiable. Third, since there was no evidence of defining time interval for detecting early OA disease course, we chose the 2 years based on the availability of follow-up data in the CHECK cohort. Further studies on exploring other time intervals or verifying this choice are needed. Fourth, a minimal amount of overfitting was detected in model 4, which might cause inaccurate probability estimations. According to a previous study, optimism is acceptable if less than 5% [40]. Therefore, we did not adjust the model intercept and coefficients.

## Conclusions

Four diagnostic models for early stage knee OA were developed based on the early clinical course and were well internally validated. Clinical course factors had statistically additive discriminative value over baseline factors, but the clinical relevance is yet to be determined. For real practice, findings of this study suggest a re-evaluation for patients with conditions get worse after baseline assessment.

## Supplementary Information

**Additional file 1.**

## Data Availability

The dataset analyzed in this paper is available from the corresponding author on reasonable request. Also, the CHECK data can be obtained from the website with request (www.check-research.com).

## Abbreviations

OA: Osteoarthritis
BMI: Body mass index
WOMAC: Western Ontario and McMaster Universities Osteoarthritis Index
JSN: Joint space narrowing
KIDA: Knee Images Digital Analysis
CI: Confidence interval
GEE: Generalized estimating equation
OR: Odds ratios
AUC: Area under the curve
TRIPOD: Transparent Reporting of a multivariable prediction model for Individual Prognosis Or Diagnosis

## References

[1] Jamshidi A, Pelletier JP, Martel-Pelletier J (2019). Machine-learning-based patient-specific prediction models for knee osteoarthritis. Nat Rev Rheumatol.

[2] Hunter DJ, Bierma-Zeinstra S (2019). Osteoarthritis. Lancet.

[3] Block JA (2014). Osteoarthritis: OA guidelines: improving care or merely codifying practice?. Nat Rev Rheumatol.

[4] Emery CA, Whittaker JL, Mahmoudian A, Lohmander LS, Roos EM, Bennell KL, Toomey CM, Reimer RA, Thompson D, Ronsky JL, Kuntze G, Lloyd DG, Andriacchi T, Englund M, Kraus VB, Losina E, Bierma-Zeinstra S, Runhaar J, Peat G, Luyten FP, Snyder-Mackler L, Risberg MA, Mobasheri A, Guermazi A, Hunter DJ, Arden NK (2019). Establishing outcome measures in early knee osteoarthritis. Nat Rev Rheumatol.

[5] Royston P, Moons KG, Altman DG, Vergouwe Y (2009). Prognosis and prognostic research: Developing a prognostic model. BMJ.

[6] Harrell FE, Lee KL, Mark DB (1996). Multivariable prognostic models: issues in developing models, evaluating assumptions and adequacy, and measuring and reducing errors. Stat Med.

[7] Sharma L, Hochberg M, Nevitt M, Guermazi A, Roemer F, Crema MD, Eaton C, Jackson R, Kwoh K, Cauley J, Almagor O, Chmiel JS (2017). Knee tissue lesions and prediction of incident knee osteoarthritis over 7 years in a cohort of persons at higher risk. Osteoarthritis Cartilage.

[8] van Oudenaarde K, Jobke B, Oostveen AC, Marijnissen AC, Wolterbeek R, Wesseling J (2017). Predictive value of MRI features for development of radiographic osteoarthritis in a cohort of participants with pre-radiographic knee osteoarthritis-the CHECK study. Rheumatology (Oxford).

[9] Kerkhof HJ, Bierma-Zeinstra SM, Arden NK, Metrustry S, Castano-Betancourt M, Hart DJ (2014). Prediction model for knee osteoarthritis incidence, including clinical, genetic and biochemical risk factors. Ann Rheum Dis.

[10] Camacho-Encina M, Balboa-Barreiro V, Rego-Perez I, Picchi F, VanDuin J, Qiu J, Fuentes M, Oreiro N, LaBaer J, Ruiz-Romero C, Blanco FJ (2019). Discovery of an autoantibody signature for the early diagnosis of knee osteoarthritis: data from the Osteoarthritis Initiative. Ann Rheum Dis.

[11] Blanco FJ, Moller I, Romera M, Rozadilla A, Sanchez-Lazaro JA, Rodriguez A (2015). Improved prediction of knee osteoarthritis progression by genetic polymorphisms: the Arthrotest Study. Rheumatology (Oxford).

[12] Lazzarini N, Runhaar J, Bay-Jensen AC, Thudium CS, Bierma-Zeinstra SMA, Henrotin Y, Bacardit J (2017). A machine learning approach for the identification of new biomarkers for knee osteoarthritis development in overweight and obese women. Osteoarthritis Cartilage.

[13] Takahashi H, Nakajima M, Ozaki K, Tanaka T, Kamatani N, Ikegawa S (2010). Prediction model for knee osteoarthritis based on genetic and clinical information. Arthritis Res Ther.

[14] Kinds MB, Marijnissen AC, Vincken KL, Viergever MA, Drossaers-Bakker KW, Bijlsma JW (2012). Evaluation of separate quantitative radiographic features adds to the prediction of incident radiographic osteoarthritis in individuals with recent onset of knee pain: 5-year follow-up in the CHECK cohort. Osteoarthritis Cartilage.

[15] Runhaar J, Kloppenburg M, Boers M, Bijlsma JWJ, Bierma-Zeinstra SMA, the CREDO expert group (2020). Towards developing diagnostic criteria for early knee osteoarthritis; data from the CHECK study. Rheumatology (Oxford).

[16] Felson D, Niu J, Sack B, Aliabadi P, McCullough C, Nevitt MC (2013). Progression of osteoarthritis as a state of inertia. Ann Rheum Dis.

[17] Bastick AN, Wesseling J, Damen J, Verkleij SP, Emans PJ, Bindels PJ (2016). Defining knee pain trajectories in early symptomatic knee osteoarthritis in primary care: 5-year results from a nationwide prospective cohort study (CHECK). Br J Gen Pract.

[18] Brand CA, Harrison C, Tropea J, Hinman RS, Britt H, Bennell K (2014). Management of osteoarthritis in general practice in Australia. Arthritis Care Res (Hoboken).

[19] Wesseling J, Boers M, Viergever MA, Hilberdink WK, Lafeber FP, Dekker J (2016). Cohort profile: Cohort Hip and Cohort Knee (CHECK) study. Int J Epidemiol.

[20] Schiphof D, Runhaar J, Waarsing JH, van Spil WE, van Middelkoop M, Bierma-Zeinstra SMA (2019). The clinical and radiographic course of early knee and hip osteoarthritis over 10 years in CHECK (Cohort Hip and Cohort Knee). Osteoarthritis Cartilage.

[21] Bellamy N, Buchanan WW, Goldsmith CH, Campbell J, Stitt LW (1988). Validation study of WOMAC: a health status instrument for measuring clinically important patient relevant outcomes to antirheumatic drug therapy in patients with osteoarthritis of the hip or knee. J Rheumatol.

[22] Marijnissen AC, Vincken KL, Vos PA, Saris DB, Viergever MA, Bijlsma JW (2008). Knee Images Digital Analysis (KIDA): a novel method to quantify individual radiographic features of knee osteoarthritis in detail. Osteoarthritis Cartilage.

[23] Kellgren JH, Lawrence JS (1957). Radiological assessment of osteo-arthrosis. Ann Rheum Dis.

[24] Runhaar J, van Middelkoop M, Reijman M, Willemsen S, Oei EH, Vroegindeweij D, van Osch G, Koes B, Bierma-Zeinstra SMA (2015). Prevention of knee osteoarthritis in overweight females: the first preventive randomized controlled trial in osteoarthritis. Am J Med.

[25] Felson DT (1996). Does excess weight cause osteoarthritis and, if so, why?. Ann Rheum Dis.

[26] McConnell S, Kolopack P, Davis AM (2001). The Western Ontario and McMaster Universities Osteoarthritis Index (WOMAC): a review of its utility and measurement properties. Arthritis Rheum.

[27] Wang Q, Runhaar J, Kloppenburg M, Boers M, Bijlsma JWJ, Bierma-Zeinstra SMA, et al. The added value of radiographs in diagnosing knee osteoarthritis is similar for general practitioners and secondary care physicians; data from the check early osteoarthritis cohort. J Clin Med. 2020;9(10):3374. 10.3390/jcm9103374 PMC7594082 33096821

[28] DeLong ER, DeLong DM, Clarke-Pearson DL (1988). Comparing the areas under two or more correlated receiver operating characteristic curves: a nonparametric approach. Biometrics.

[29] Moons KG, Altman DG, Reitsma JB, Ioannidis JP, Macaskill P, Steyerberg EW (2015). Transparent Reporting of a multivariable prediction model for Individual Prognosis or Diagnosis (TRIPOD): explanation and elaboration. Ann Intern Med.

[30] Baker SG, Schuit E, Steyerberg EW, Pencina MJ, Vickers A, Moons KGM, Mol BWJ, Lindeman KS (2014). How to interpret a small increase in AUC with an additional risk prediction marker: decision analysis comes through. Stat Med.

[31] Wesseling J, Dekker J, van den Berg WB, Bierma-Zeinstra SM, Boers M, Cats HA (2009). CHECK (Cohort Hip and Cohort Knee): similarities and differences with the Osteoarthritis Initiative. Ann Rheum Dis.

[32] Segal NA, Nevitt MC, Gross KD, Hietpas J, Glass NA, Lewis CE, Torner JC (2013). The Multicenter Osteoarthritis Study: opportunities for rehabilitation research. PM R.

[33] Mallen CD, Peat G, Thomas E, Lacey R, Croft P (2007). Predicting poor functional outcome in community-dwelling older adults with knee pain: prognostic value of generic indicators. Ann Rheum Dis.

[34] Alba AC, Agoritsas T, Walsh M, Hanna S, Iorio A, Devereaux PJ, McGinn T, Guyatt G (2017). Discrimination and calibration of clinical prediction models: users’ guides to the medical literature. JAMA.

[35] Crema MD, Nevitt MC, Guermazi A, Felson DT, Wang K, Lynch JA, Marra MD, Torner J, Lewis CE, Roemer FW (2014). Progression of cartilage damage and meniscal pathology over 30 months is associated with an increase in radiographic tibiofemoral joint space narrowing in persons with knee OA--the MOST study. Osteoarthritis Cartilage.

[36] Wesseling J, Bierma-Zeinstra SM, Kloppenburg M, Meijer R, Bijlsma JW (2015). Worsening of pain and function over 5 years in individuals with ‘early’ OA is related to structural damage: data from the Osteoarthritis Initiative and CHECK (Cohort Hip & Cohort Knee) study. Ann Rheum Dis.

[37] B OH, Gransar H, Callister T, Shaw LJ, Schulman-Marcus J, Stuijfzand WJ, et al. (2018). Development and validation of a simple-to-use nomogram for predicting 5-, 10-, and 15-year survival in asymptomatic adults undergoing coronary artery calcium scoring. JACC Cardiovasc Imaging.

[38] Deyle GD, Allen CS, Allison SC, Gill NW, Hando BR, Petersen EJ, Dusenberry DI, Rhon DI (2020). Physical therapy versus glucocorticoid injection for osteoarthritis of the knee. N Engl J Med.

[39] Nelson AE, Allen KD, Golightly YM, Goode AP, Jordan JM (2014). A systematic review of recommendations and guidelines for the management of osteoarthritis: the chronic osteoarthritis management initiative of the U.S. bone and joint initiative. Semin Arthritis Rheum.

[40] Riley RD, Ensor J, Snell KIE, Harrell FE, Martin GP, Reitsma JB (2020). Calculating the sample size required for developing a clinical prediction model. BMJ.

