# Supplementary material for: Diagnosis of early stage knee osteoarthritis based on early clinical course: data from the CHECK cohort
Source: Arthritis Res Ther. 2021 Aug 19;23:217. doi: 10.1186/s13075-021-02598-5 (PMC8375192; doi:10.1186/s13075-021-02598-5)
Supplement: Supplementary file 1 — Additional file 1. [file 13075_2021_2598_MOESM1_ESM.docx]

**Appendix table1.** Internal validation of model over-fitting.

|  | Original AUC | Corrected AUC | Optimism (%) |
| --- | --- | --- | --- |
| Model 1 | 0.70 | 0.70 | 0 |
| Model 2 | 0.74 | 0.74 | 0 |
| Model 3 | 0.77 | 0.77 | 0 |
| Model 4 | 0.80 | 0.79 | 1 |

Model 1, included clinical course factors only; model 2, included clinical and radiographic course factors; model 3, clinical course factors + clinical baseline factors; model 4, clinical and radiographic course factors + clinical and radiographic baseline factors.

AUC, area under the curve.

|  | Pooled AUC | | | Difference  (%) |
| --- | --- | --- | --- | --- |
|  | Original | ‘uncertain’ as ‘OA’ | ‘uncertain’ as ‘no OA’ |  |
| Model 1 | 0.70 | 0.68 | 0.67 | -3, -4 |
| Model 2 | 0.74 | 0.72 | 0.70 | -3, -5 |
| Model 3 | 0.77 | 0.74 | 0.72 | -4, -6 |
| Model 4 | 0.80 | 0.77 | 0.75 | -4, -6 |

**Appendix table 2.** Sensitivity analysis by including ‘uncertain’ knees.

Model 1, included clinical course factors only; model 2, included clinical and radiographic course factors; model 3, clinical course factors + clinical baseline factors; model 4, clinical and radiographic course factors + clinical and radiographic baseline factors.

AUC, area under the curve.

**Appendix figure.** Factors’ contributions in 4 models. The image presents the changes in area under the curve while doing backward selection. The most significant factors are presented at the tops of plots; – course means 2-year course factors; WOMAC, Western Ontario and McMaster Universities Osteoarthritis Index; TF, tibia femoral; JSN, joint space narrowing; PF, patellofemoral joint. Model 1, included clinical course factors only; model 2, included clinical and radiographic course factors; model 3, clinical course factors + clinical baseline factors; model 4, clinical and radiographic course factors + clinical and radiographic baseline factors.

**
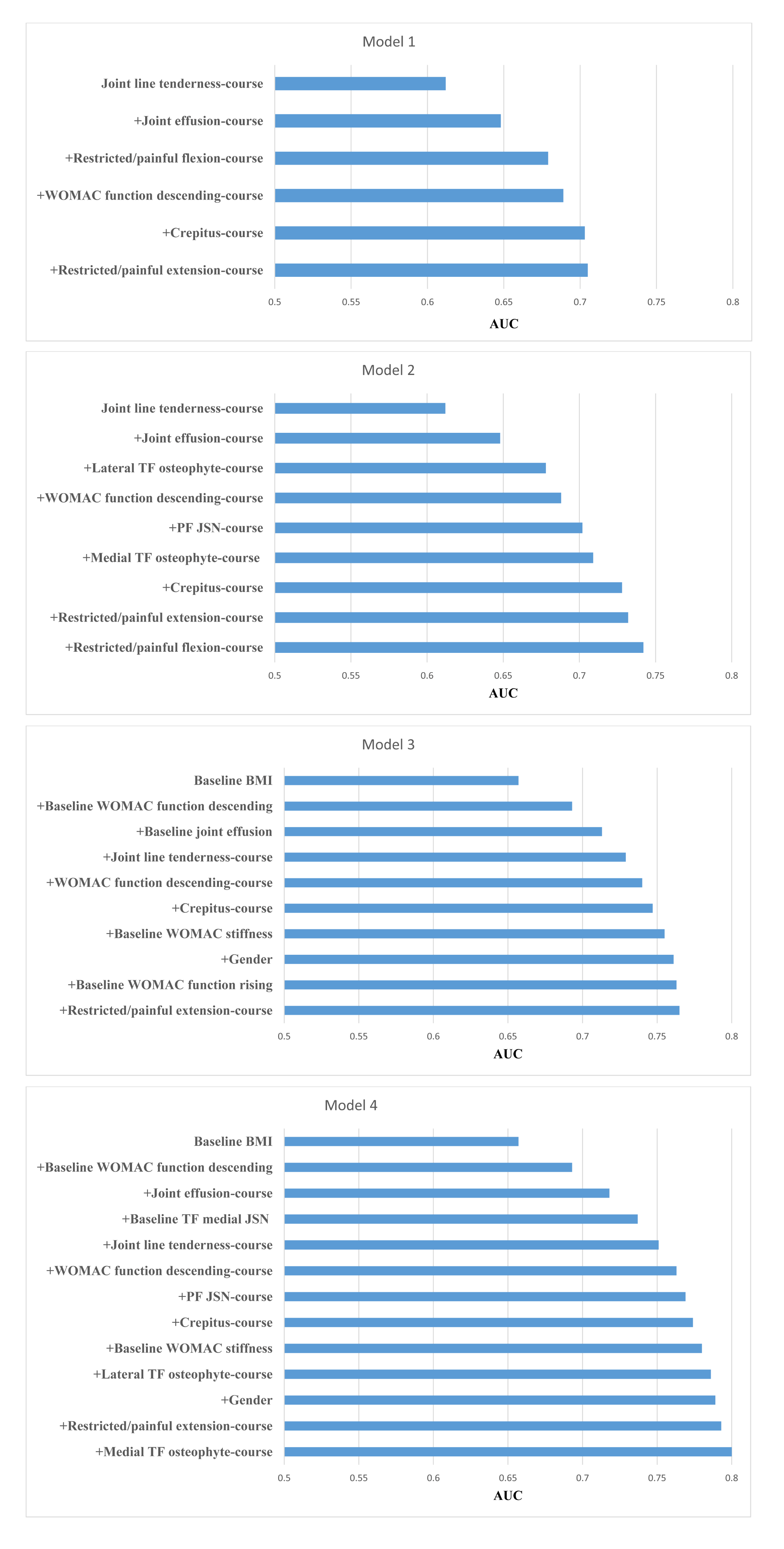
**
